# Supplementary figures and images for: The EARP Complex and Its Interactor EIPR-1 Are Required for Cargo Sorting to Dense-Core Vesicles
Source: PLoS Genet. 2016 May 18;12(5):e1006074. doi: 10.1371/journal.pgen.1006074 (PMC4871572; doi:10.1371/journal.pgen.1006074)

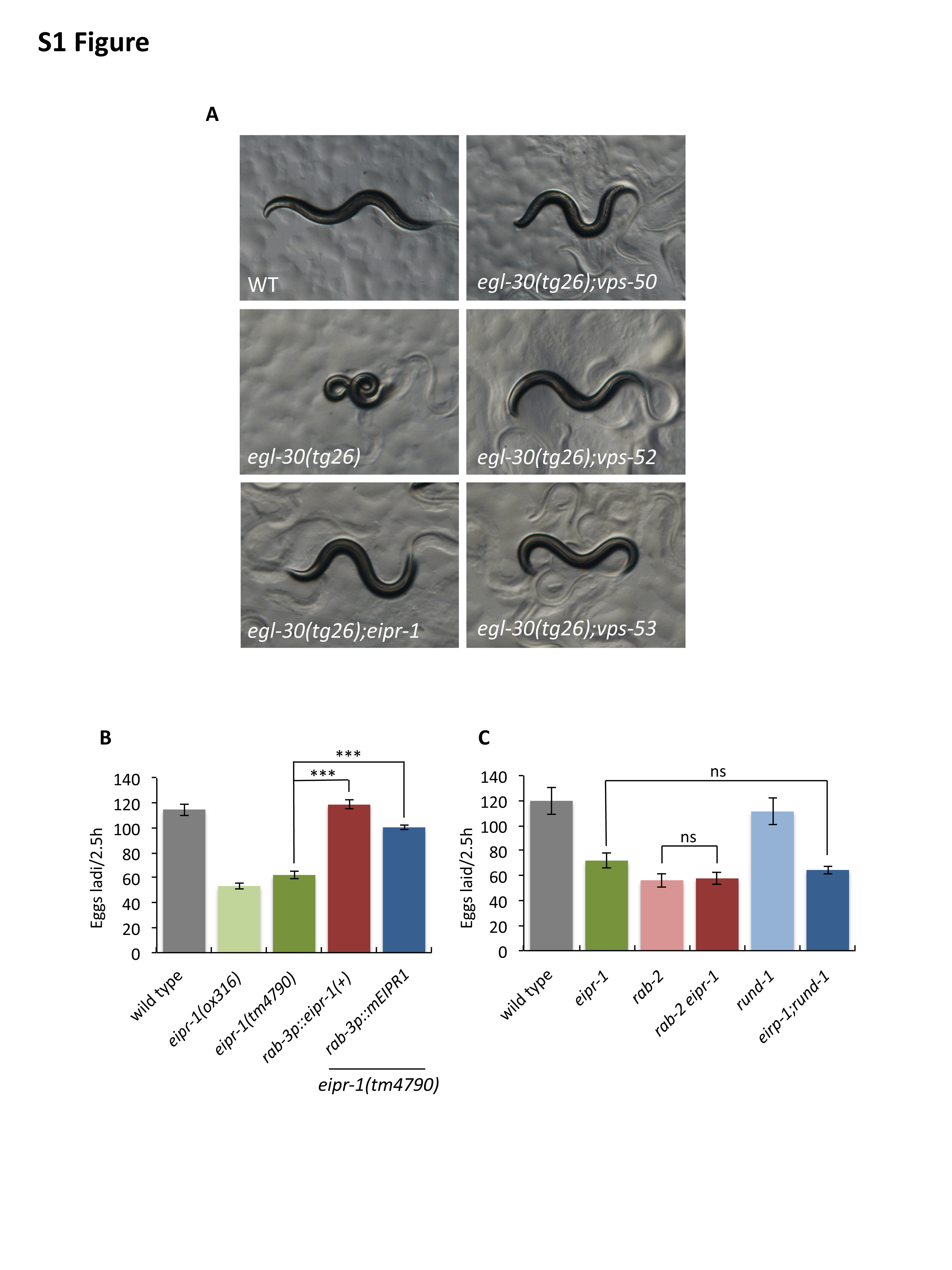

Supplement: S1 Fig — (A) The activated Gq mutant egl-30(tg26) has hyperactive locomotion and deep body bends. The egl-30(tg26) locomotion phenotype is suppressed by the eipr-1(ox316), vps-50(ok2627), vps-52(ox345), and vps-53(ox339) mutations. (B) eipr-1 acts in neurons to control egg laying. The graph shows the number of eggs laid by 5 animals in a 2.5 hour period. eipr-1 mutants show egg-laying defects and this phenotype is rescued by panneuronal expression of either the worm gene or its mouse ortholog (***, P<0.001). Error bars = SEM; n = 10 plates of 5 worms each. (C) eipr-1 acts in the same pathway as rab-2 to control egg-laying. Double mutants of eipr-1(tm4790) with rab-2(nu415) or rund-1(tm3622) do not have stronger egg-laying defects than the single mutants. Though rund-1(tm3622) mutants have a visible Egl (egg-laying defective) phenotype indicating that they retain more eggs, they did not have a significantly reduced egg-laying rate as measured by this assay. Error bars = SEM; n = 5 plates of 5 worms each. ns, not significant, P>0.05. (TIF) [file pgen.1006074.s001.tif]

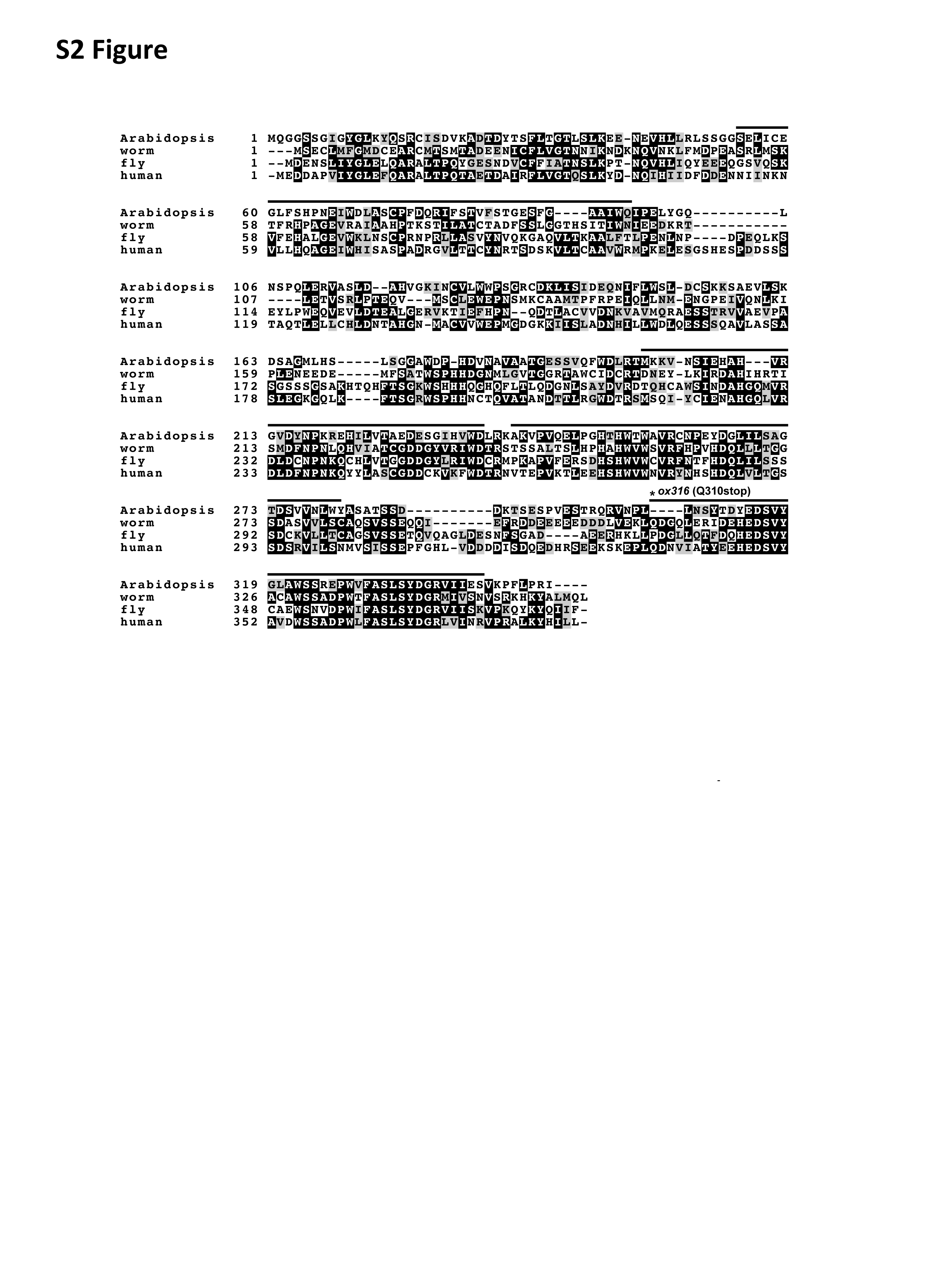

Supplement: S2 Fig — Alignment of C. elegans EIPR-1 (worm, accession # NP_493383.1) and its orthologs from Arabidopsis thaliana (Arabidopsis, accession # NP_173478.2), Drosophila melanogaster (fly, CG10646, accession # NP_648581.1), and Homo sapiens (human, TSSC1, accession # NP_003301.1). Identical residues are shaded in black and similar residues are shaded in gray. The WD40 repeats (from SMART, using the worm protein; http://smart.embl-heidelberg.de/) are marked with single black bars. Using SMART, worm EIPR-1 has four predicted WD40 repeats, Arabidopsis has six, fly has three, and human has five. WD40 repeats are difficult to identify by primary sequence and are often missed by prediction programs. The position of the ox316 stop mutation is marked with an asterisk. Alignment was made with MUSCLE (http://www.ebi.ac.uk/Tools/msa/muscle/) using default parameters and exhibited with Boxshade 3.21 (http://www.ch.embnet.org/software/BOX_form.html). (TIF) [file pgen.1006074.s002.tif]

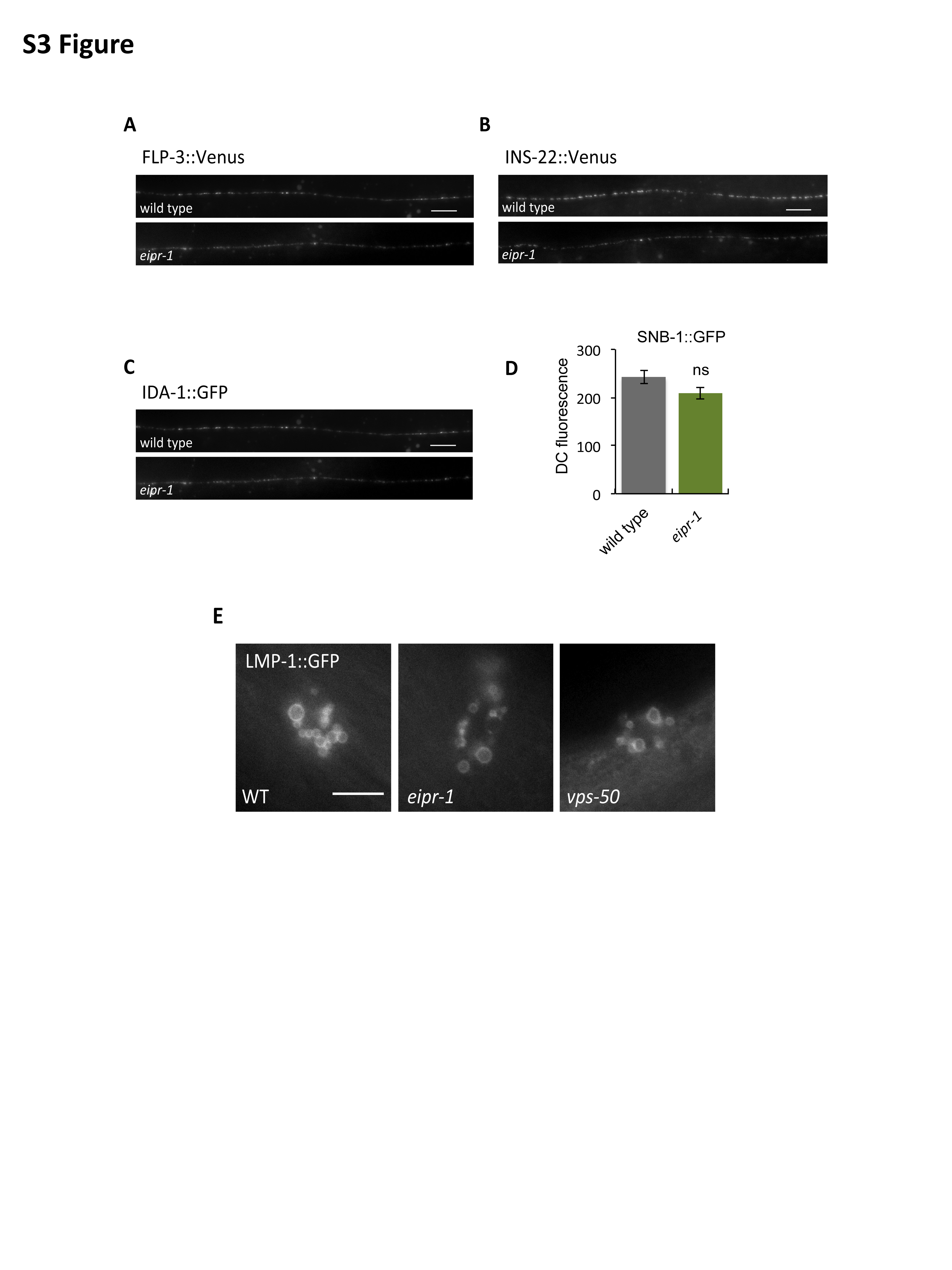

Supplement: S3 Fig — (A) Representative images of FLP-3::Venus (ceIs61) fluorescence in dorsal cord motor neuron axons of the wild type and eipr-1(tm4790) mutant strains. Scale bar: 10 μm. eipr-1(tm4790) mutants have decreased fluorescence in the dorsal cord, indicative of a FLP-3::Venus sorting or trafficking defect. (B) Representative images of INS-22::Venus (nuIs195) fluorescence in dorsal cord motor neuron axons. Scale bar: 10 μm. (C) Representative images of IDA-1::GFP (ceIs72) fluorescence in the dorsal nerve cord. Scale bar: 10 μm. (D) Levels of the synaptic vesicle cargo synaptobrevin, SNB-1::GFP (nuIs152), in the dorsal nerve cord. eipr-1(tm4790) mutants do not have a defect (ns = not significant, P>0.05). Error bars = SEM; n = 21–26. (E) eipr-1 and vps-50 mutants do not have enlarged lysosomes. Representative images of LMP-1::GFP (pwIs50) fluorescence in coelomocytes of wild-type (WT), eipr-1(tm4790) and vps-50(ok2627) mutant strains. Scale bar: 10 μm. (TIF) [file pgen.1006074.s003.tif]

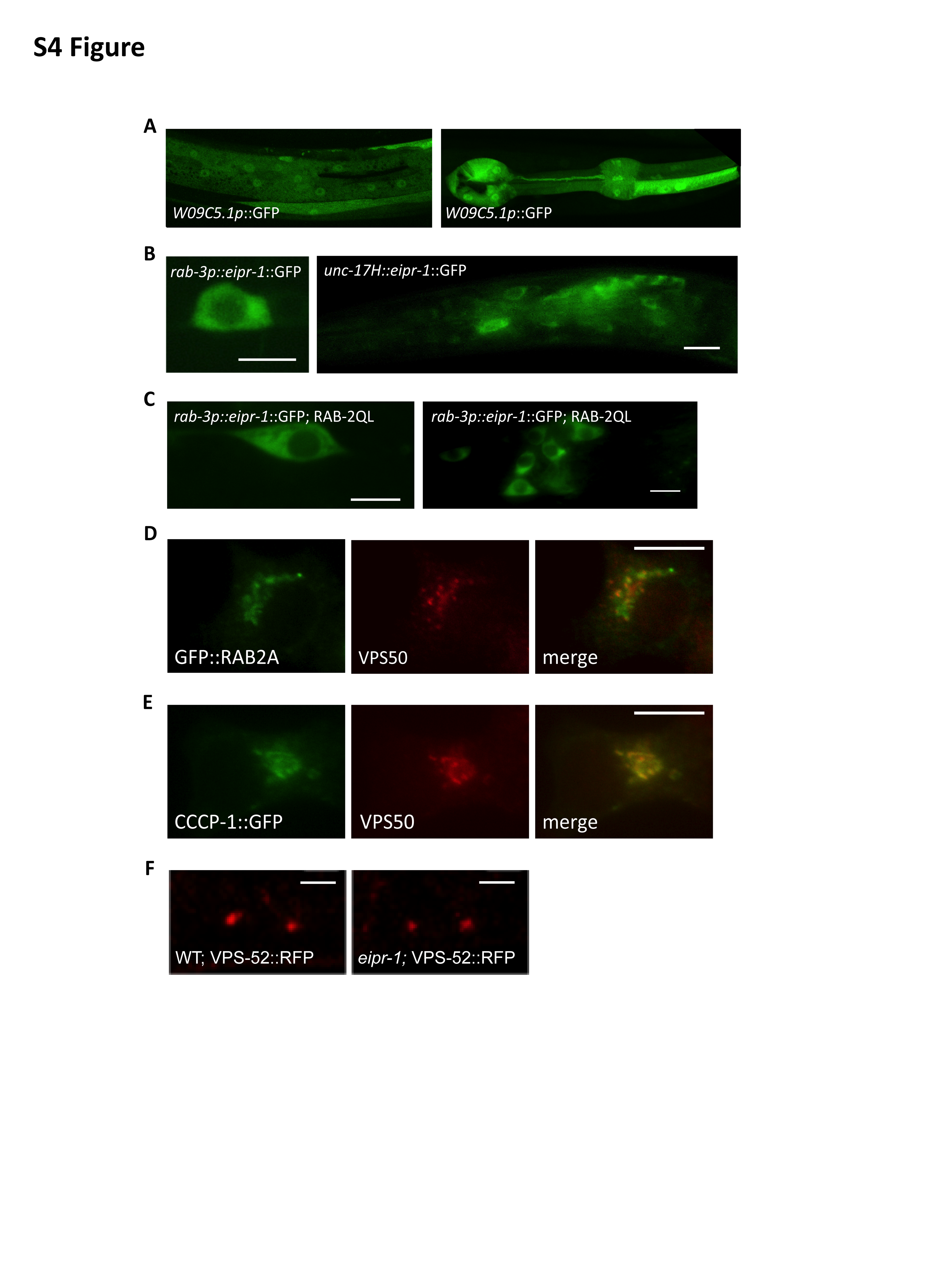

Supplement: S4 Fig — (A) Representative images of animals expressing GFP under the promoter region upstream of the first gene (W09C5.1) of the operon where eipr-1 is located. The image on the left shows expression in the hypodermis (skin) while the image on the right shows expression in the pharynx. (B) Representative images of neurons expressing eipr-1::GFP under panneuronal (Left panel, rab-3p) and head cholinergic (Right panel, unc-17Hp) promoters. Scale bars: 5 μm. (C) Representative images of neurons coexpressing rab-3p::eipr-1::GFP and GTP-bound RAB-2 (RAB-2QL). Scale bars: 5 μm. (D) Representative images of 832/13 cells expressing GFP::RAB2A and costained for endogenous VPS50. Scale bar: 10 μm. VPS50 partially colocalizes with RAB2A. (E) Representative images of 832/13 cells expressing CCCP1::GFP and costained for endogenous VPS50. Scale bar: 10 μm. VPS50 largely colocalizes with CCCP1. (F) Representative images of wild-type (WT) and eipr-1(tm4790) mutant neurons expressing VPS-52::tagRFP. Scale bars: 1 μm. VPS-52 localizes normally in eipr-1 mutants. (TIF) [file pgen.1006074.s004.tif]

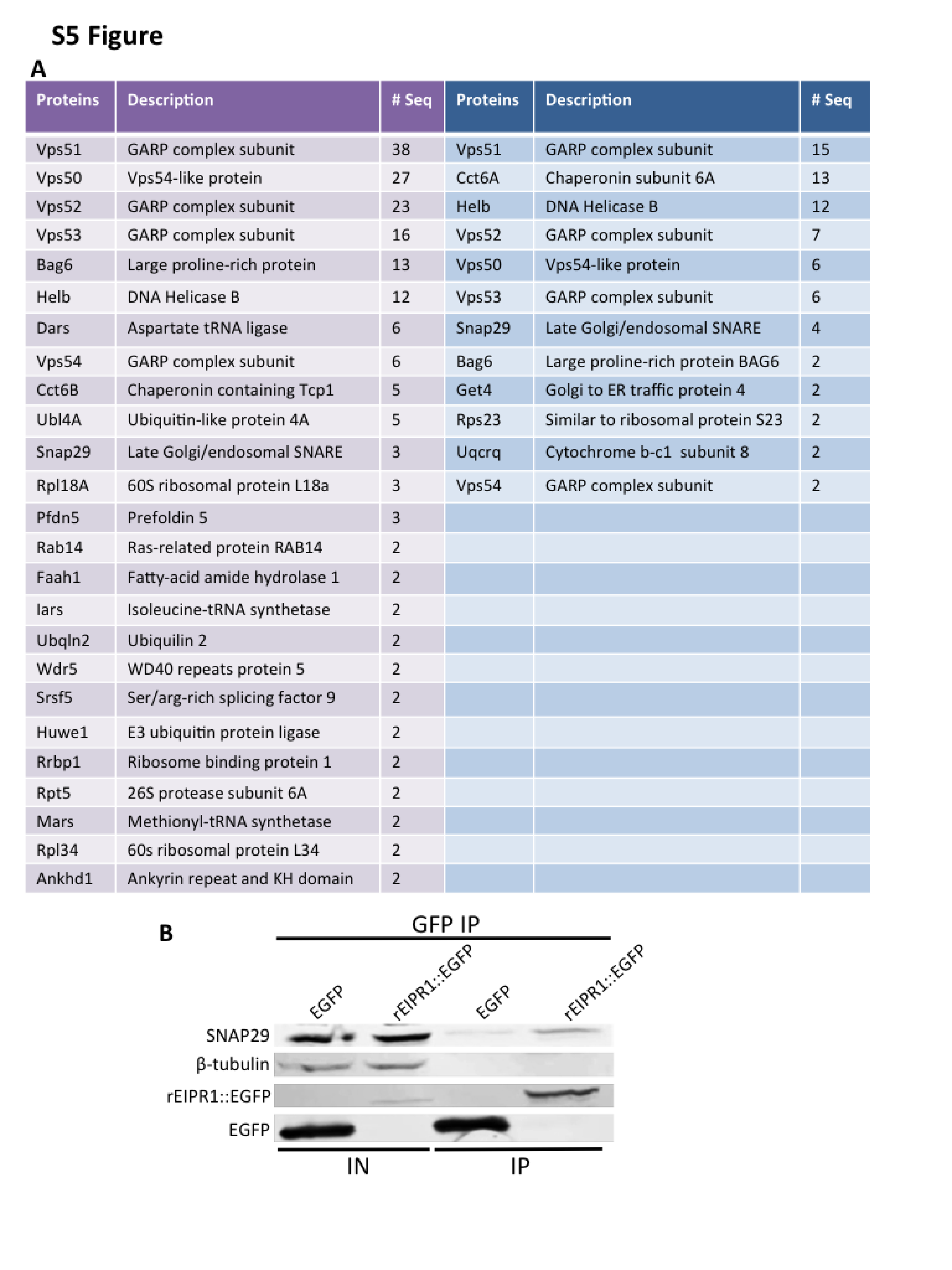

Supplement: S5 Fig — (A) List of top hits from two independent experiments performing mass spectrometry of a pulldown of rEIPR1::GFP in 832/13 cells. On the left side of the table, we show the list of hits found in a mass spec experiment after subtracting hits found in a GFP control pulldown. On the right side of the table, we show the list of hits found in an independent mass spec experiment after subtracting hits found in either the pulldown of CCCP1::GFP or RUNDC1::GFP. In both cases, EIPR1::GFP pulldowns were performed in parallel to controls. # seq = number of unique peptides from each protein. All proteins with two or more unique peptides are shown. (B) EGFP-tagged rat EIPR1 or EGFP was expressed in 832/13 cells. Immunoprecipitation of EIPR1::EGFP pulled down more SNAP29 than untagged EGFP. IN: input; IP: immunoprecipitation. (TIF) [file pgen.1006074.s005.tif]

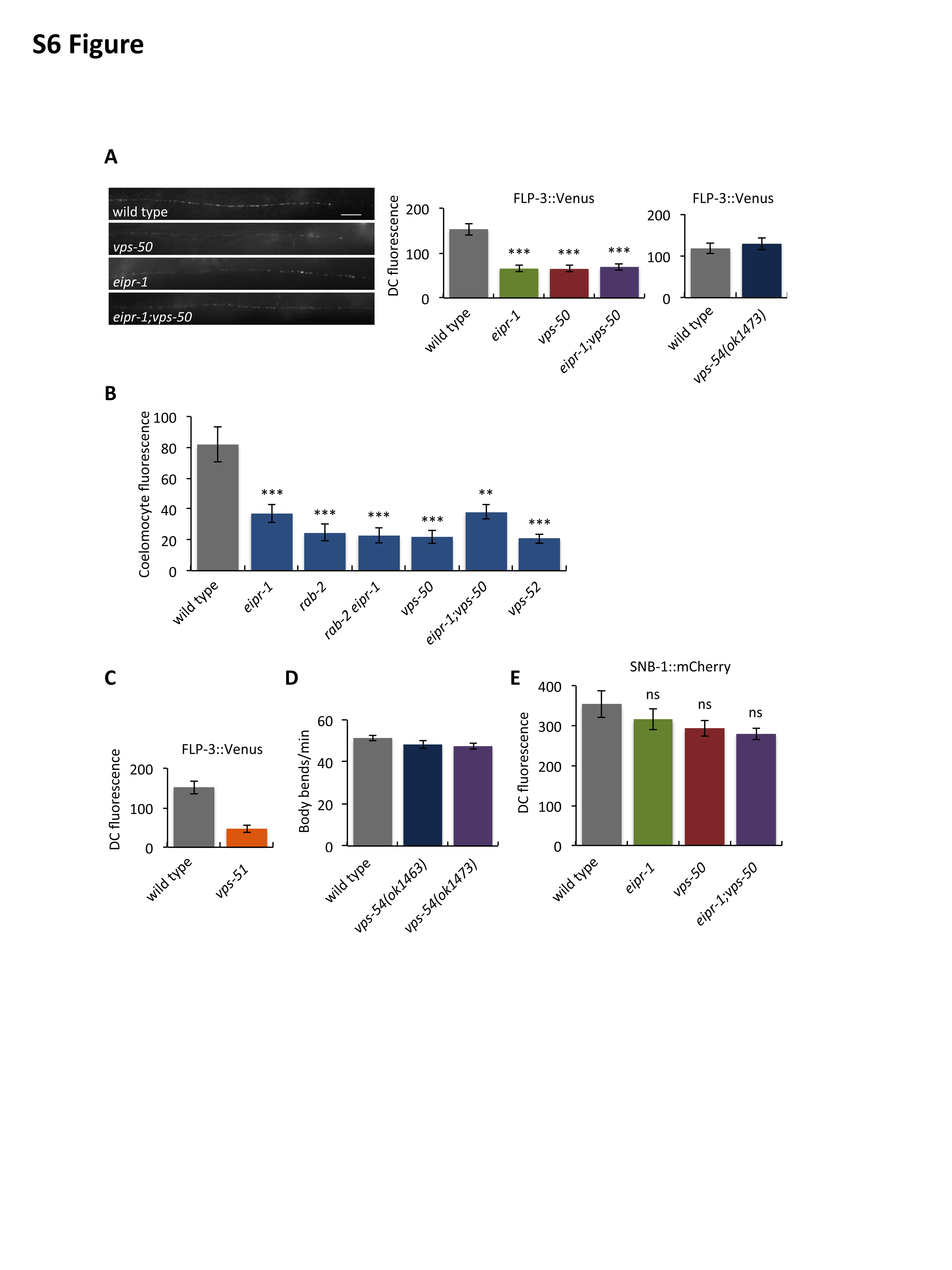

Supplement: S6 Fig — (A) vps-50 acts in the same genetic pathway as eipr-1 to control FLP-3::Venus trafficking. Left: representative images. Right: quantification. vps-50(ok2627) and eipr-1(tm4790) have reduced FLP-3::Venus (ceIs61) fluorescence, but an eipr-1(tm4790); vps-50(ok2627) double mutant does not have a stronger phenotype than either single mutant indicating that eipr-1 and vps-50 act in the same genetic pathway. A vps-54(ok1473) mutant does not have decreased axonal levels of FLP-3::Venus. ***, P<0.001 compared to wild type. Error bars = SEM; n = 9–14. (B) eipr-1 and GARP/EARP mutants have reduced secretion of NLP-21::Venus. NLP-21::Venus (nuIs183) fluorescence levels in the coelomocytes of the indicated strains. Like rab-2(nu415) mutants, eipr-1(tm4790), vps-50(ok2627), and vps-52(ox345) mutants show reductions in the accumulation of Venus fluorescence in coelomocytes that is approximately proportional to the decrease in axonal fluorescence seen in these mutants, suggesting that these mutants are not defective in dense-core vesicle release. Double mutants between eipr-1(tm4790) and rab-2(nu415) or vps-50(ok2627) do not have stronger phenotypes than the single mutants, suggesting that these genes all act in the same dense-core vesicle cargo sorting pathway. **, P<0.01; ***, P<0.001 compared to WT. Error bars = SEM; n = 14–33. (C) vps-51 mutants have defects in trafficking FLP-3::Venus. FLP-3::Venus (ceIs61) fluorescence levels in the dorsal nerve cord of wild type and vps-51(tm4275) mutant strains. Error bars = SEM; n = 8. (D) GARP mutants vps-54(ok1463) and vps-54(ok1473) do not have a reduced locomotion rate. Error bars = SEM; n = 10. (E) eipr-1(tm4790) and vps-50(ok2627) mutants do not have defects in trafficking synaptic vesicle cargos. SNB-1::mCherry (ceIs61) fluorescence levels were measured in the dorsal nerve cord. ns, not significant, P>0.05 compared to wild type. Error bars = SEM; n = 11–14. (TIF) [file pgen.1006074.s006.tif]
