# Supplementary material for: The EARP Complex and Its Interactor EIPR-1 Are Required for Cargo Sorting to Dense-Core Vesicles
Source: PLoS Genet. 2016 May 18;12(5):e1006074. doi: 10.1371/journal.pgen.1006074 (PMC4871572; doi:10.1371/journal.pgen.1006074)
Supplement: S1 Table — (DOCX) [file pgen.1006074.s007.docx]

**Table S1. List of strains**

*nuIs152[Punc-129::GFP::SNB-1, Pttx-3::RFP] II*^a^

CB4856 Hawaiian wild isolate

EG316 *eipr-1(ox316mat) I*

EG339 *vps-53(ox339mat) III*

EG345 *vps-52(ox345) X*

EG476 *vps-50(ox476) III*

EG3635 *egl-30(tg26) I eipr-1(ox316mat) I*

EG3746 *egl-30(tg26) I ; vps-53(ox339mat) III*

EG3870 *egl-30(tg26) I ; vps-52(ox345) X*

EG4258 *eipr-1(ox316mat) I* ; *oxEx1106[RPCI94_01L06, Pmyo-2::gfp, lin-15(+)]*

EG5246 *eipr-1(ox316mat) I ; nuIs183[Punc-129::NLP-21-Venus, Pmyo-2::NLS-GFP] III*

EG5281 *nuIs183[Punc-129::NLP-21-Venus, Pmyo-2::NLS-GFP] III ; vps-52(ox345) X*

EG5335 *ceIs61[Punc-129::flp-3::venus, Punc-129::mCherry-snb-1, Pttx-3::mCherry] II ; vps-53(ox339mat) III*

EG5401 *ceIs61[Punc-129::flp-3::venus, Punc-129::mCherry-snb-1, Pttx-3::mCherry] II ; vps-52(ox345) X*

EG5505 *rund-1(tm3622) X*^b^

EG5627 *rab-2(nu415) I*^b^

EG5674 *nuIs183[Punc-129::NLP-21::Venus, Pmyo-2::NLS-GFP] III* ; *rund-1(tm3622) X*^b^

EG5690 *vps-54(ok1463) V*

EG5699 *vps-52(ok853) X*

EG5700 *vps-53(ok2864) III*

EG5714 *vps-54(ok1473) V*

EG5806 *ceIs61[Punc-129::flp-3::venus, Punc-129::mCherry-snb-1, Pttx-3::mCherry] II ; vps-54(ok1473) V*

EG5828 *lin-15(n765ts) X ; oxEx1445[Pvps-52::citrine, lin-15(+)]*

EG5829 *lin-15(n765ts) X ; oxEx1446[Pvps-53::citrine, lin-15(+)]*

EG5854 *vps-53(ox339mat) III ; oxEx1449[Pvps-53::vps-53(+)::tagRFP, Pmyo-2::mCherry, Pmyo-3::mCherry, Prab-3::mCherry]*

EG5855 *vps-53(ox339mat) III ; oxEx1450[Prab-3::vps-53(+)::tagRFP, Pmyo-2::mCherry, Pmyo-3::mCherry, Prab-3::mCherry]*

EG5856 *vps-52(ox345) X ; oxEx1451[Pvps-52::vps-52(+)::tagRFP, Pmyo-2::GFP]*

EG5920 *vps-53(ox339mat) III ; ceIs72[Punc-129::ida-1::GFP, Pttx-3::mCherry] IV*

EG5929 *ceIs72[Punc-129::ida-1::GFP, Pttx-3::mCherry] IV ; vps-52(ox345) X*

EG5963 *oxSi190[Pvps-53::vps-53(+)::tagRFP, Cbunc-119(+)] II ; unc-119(ed3) III*

EG5965 *oxSi192[Pvps-52::vps-52(+)::tagRFP, Cbunc-119(+)] II ; unc-119(ed9) III*

EG6007 *vps-50(ok2627) III*

EG6009 *vps-50(ok2627) III ; ceIs72[Punc-129::ida-1::GFP, Pttx-3::mCherry] IV* EG6037 *vps-51(tm4275) I ; ceIs61[Punc-129::flp-3::venus, Punc-129::mCherry-snb-1, Pttx-3::mCherry] II*

EG6916 *eipr-1(ox316mat) I ; oxIs590[Cb unc-119(+), Prund-1::rund-1(+)::tagRFP] II*

EG6919 *eipr-1(ox316mat) I ; oxSi314[Prab-2::eGFP::rab-2(+), Cb-unc-119] IV*

EG6939 *eipr-1(tm4790)* *I*

EG8116 *oxSi702[unc-17::GFP, unc-119(+)] II*^c^

KG1395 *nuIs183[Punc-129::NLP-21::Venus, Pmyo-2::NLS-GFP] III*^d^

KG1624 *nuIs195[Punc-129::INS-22::Venus, Pmyo-2::GFP] IV*^d^

KG1645 *ceIs61[Punc-129::FLP-3::Venus, Punc-129::mCherry-SNB-1, Pttx-3::mCherry] II*^d^

KG1852 *ceIs72[Punc-129::IDA-1::GFP, Pttx-3::mCherry] IV*^d^

MT15894 *vps-50(n4022) III*^e^

N2 Bristol isolate, standard lab wild type

RT258 *unc-119(ed3) III ; pwIs50[lmp-1::GFP + Cb-unc-119(+)]*^f^

XZ1026 *rab-2(nu415) I ; nuIs183[Punc-129::NLP-21::Venus, Pmyo-2::NLS-GFP] III*^b^

XZ1039 *eipr-1(ox316mat) I ; oxEx1390[Prund-1::eipr-1(+)::tagRFP, Pmyo-2::gfp]*

XZ1040 *eipr-1(ox316mat) I ; oxEx1249[Prab-3::eipr-1(+) cDNA::GFP, myo-2::mCherry]*

XZ1053 *eipr-1(tm4790)* *I ; nuIs183[Punc-129::NLP-21::Venus, Pmyo-2::NLS-GFP] III* ; *rund-1(tm3622) X*

XZ1055 *eipr-1(tm4790) I ; nuIs183[Punc-129::NLP-21::Venus, Pmyo-2::NLS-GFP] III*

XZ1109 *eipr-1(tm4790) I ; yakEx1[Prab-3::eipr-1(+)::GFP, Pmyo-3::mCherry]*

XZ1124 *egl-30(tg26) I eipr-1(tm4790) I*

XZ1145 *vps-51(tm4275) I*

XZ1151 *egl-30(tg26) I*

XZ1178 *eipr-1(tm4790) I ; ceIs61[Punc-129::FLP-3::Venus, Punc-129::mCherry-SNB-1, Pttx-3::mCherry] II*

XZ1184 *eipr-1(tm4790) I ; yakSi2[Punc-17H::eipr-1(+)::GFP, cb-unc-119(+)] II; unc-119(ed3) III*

XZ1282 *eipr-1(tm4790) I ; nuIs152[Punc-129:: GFP::SNB-1, Pttx-3::RFP] II*

XZ1289 *eipr-1(tm4790)* *I ; nuIs183[Punc-129::NLP-21::Venus, Pmyo-2::NLS-GFP] III* ; *egl-3(ok979) V*

XZ1290 *nuIs183[Punc-129::NLP-21::Venus, Pmyo-2::NLS-GFP] III* ; *egl-3(ok979) V*

XZ1318 *eipr-1(tm4790) I ; ceIs72[Punc-129:: IDA-1::GFP, Pttx-3::mCherry] IV*

XZ1320 *eipr-1(tm4790) I nuIs195[Punc-129::INS-22::Venus, Pmyo-2::GFP] IV*

XZ1321 *eipr-1(tm4790) I ; yakSi4[Prab-3::eipr-1(+)::GFP, cb-unc-119(+)] II; unc-119(ed3) III*

XZ1338 *eipr-1(tm4790) I ; oxSi702[unc-17::GFP, unc-119(+)] II*

XZ1348 *eipr-1(tm4790) I ; yakSi5[Punc-17::eipr-1(+)::GFP, cb-unc-119(+)] II; unc-119(ed3) III*

XZ1386 *eipr-1(tm4790) I ; yakSi10[Pacr-2::eipr-1(+)::GFP, cb-unc-119(+)] II; unc-119(ed3) III*

XZ1415 *eipr-1(tm4790) I ; yakSi4[Prab-3::eipr-1(+)::GFP,cb-unc-119(+)] II; nuIs183[Punc-129::NLP-21::Venus, Pmyo-2::NLS-GFP] III*

XZ1416 *eipr-1(tm4790) I ; yakSi2[Punc-17H::eipr-1(+)::GFP, cb-unc-119(+)] II; unc-119(ed3) III ; nuIs183[Punc-129::NLP-21::Venus, Pmyo-2::NLS-GFP] III*

XZ1474 *rab-2(nu415) I eipr-1(tm4790)* *I ; nuIs183[Punc-129::NLP-21::Venus, Pmyo-2::NLS-GFP] III*

XZ1484 *eipr-1(tm4790) I ; yakEx38[Prab-3::mEIPR1::GFP, Pmyo-3::mCherry]*

XZ1594 *eipr-1(tm4790) I ; vps-50(ok2627) III*

XZ1596 *eipr-1(tm4790) I ; ceIs61[Punc-129::FLP-3::Venus, Punc-129::mCherry-SNB-1, Pttx-3::mCherry] II ; vps-50(ok2627) III*

XZ1604 *ceIs72[Punc-129::ida-1::GFP, Pttx-3::mCherry] IV ; vps-54(ok1463) V*

XZ1607 *nuIs183[Punc-129::NLP-21-Venus, Pmyo-2::NLS-GFP] III vps-50(ok2627) III* XZ1614 *egl-30(tg26) I ; vps-50(ok2627) III*

XZ1632 *vps-50(n4022) III ; ceIs72[Punc-129::ida-1::GFP, Pttx-3::mCherry] IV*

XZ1634 *eipr-1(tm4790) I ; vps-50(ok2627) III nuIs183[Punc-129::NLP-21::Venus, myo-2::NLS-GFP] III*

XZ1640 *eipr-1(tm4790) I ; nuIs183[Punc-129::NLP-21-Venus, Pmyo-2::NLS-GFP] III ; yakEx70[Punc-129::eipr-1::GFP; Pmyo-2:mCherry]*

XZ1647 *eipr-1(tm4790) I ; oxSi192[Pvps-52::vps-52(+)::tagRFP, Cbunc-119(+)] II*

XZ1649 *vps-51(tm4275) I ; nuIs183[Punc-129::NLP-21-Venus, Pmyo-2::NLS-GFP] III*

XZ1653 *vps-51(tm4275) I ; ceIs72[Punc-129::ida-1::GFP, Pttx-3::mCherry] IV*

XZ1660 *eipr-1(tm4790) I ; pwIs50[lmp-1::GFP, Cb-unc-119(+)]*

XZ1661 *vps-50(ok2627) III ; pwIs50[lmp-1::GFP + Cb-unc-119(+)]*

XZ1670 *nuIs183[Punc-129::NLP-21-Venus, Pmyo-2::NLS-GFP] III ; vps-54(ok1463) V*

XZ1671 *egl-30(tg26) I ; vps-54(ok1463) V*

XZ1746 *oxSi192[Pvps-52::vps-52(+)::tagRFP, Cb-unc-119(+)] II ; oxSi314[Prab-2::eGFP::rab-2, Cb-unc-119] IV*

XZ1748 *oxSi190[Pvps-53::vps-53(+)::tagRFP, Cb-unc-119(+)] II ; oxSi314[Prab-2::eGFP::rab-2, Cb-unc-119] IV*

XZ1750 *rab-2(nu415) I ; vps-53(ox339mat) III*

XZ1752 *rab-2(nu415) I ;* *ceIs61[Punc-129::FLP-3::Venus, Punc-129::mCherry-SNB-1, Pttx-3::mCherry] II; vps-53(ox339mat) III*

All non-wild strains were produced in this study except for the following referenced strains:

^a^ strain was a gift from J. Bai. transgene *nuIs152* was first described in Sieburth et al. (2005)

^b^ from Ailion et al. (2014)

^c^ gift from M. Schwartz

^d^ from Edwards et al. (2009). transgenes *nuIs183* and *nuIs195* were first described in Sieburth et al. (2007)

^e^ from Paquin et al. (2016)

^f^ from Treusch et al. (2004)

**References**

Ailion M, Hannemann M, Dalton S, Pappas A, Watanabe S, Hegermann J, et al. Two Rab2 interactors regulate dense-core vesicle maturation. Neuron. 2014 Apr 2;82(1):167–80.

Edwards SL, Charlie NK, Richmond JE, Hegermann J, Eimer S, Miller KG. Impaired dense core vesicle maturation in Caenorhabditis elegans mutants lacking Rab2. J Cell Biol. 2009 Sep 21;186(6):881–95.

Paquin N, Murata Y, Froehlich A, Omura DT, Ailion M, Pender CL, et al. The Conserved VPS-50 Protein Functions in Dense-Core Vesicle Maturation and Acidification and Controls Animal Behavior. Curr Biol. 2016 Apr 4;26(7):862–71.

Sieburth D, Ch’ng Q, Dybbs M, Tavazoie M, Kennedy S, Wang D, et al. Systematic analysis of genes required for synapse structure and function. Nature. 2005 Jul 28;436(7050):510–7.

Sieburth D, Madison JM, Kaplan JM. PKC-1 regulates secretion of neuropeptides. Nat Neurosci. 2007 Jan;10(1):49–57.

Treusch S, Knuth S, Slaugenhaupt SA, Goldin E, Grant BD, Fares H. Caenorhabditis elegans functional orthologue of human protein h-mucolipin-1 is required for lysosome biogenesis. Proc Natl Acad Sci USA. 2004 Mar 30;101(13):4483–8.
