# Supplementary material for: The EARP Complex and Its Interactor EIPR-1 Are Required for Cargo Sorting to Dense-Core Vesicles
Source: PLoS Genet. 2016 May 18;12(5):e1006074. doi: 10.1371/journal.pgen.1006074 (PMC4871572; doi:10.1371/journal.pgen.1006074)
Supplement: S2 Table — (DOCX) [file pgen.1006074.s008.docx]

**Table S2. List of plasmids**

Gateway destination vectors

pCFJ150 Gateway destination vector for insertion at chromosome II Mos site *ttTi5605*

Gateway entry clones

pADA180 *unc-17Hp* [4-1] (head cholinergic neurons only)

pCFJ31 *acr-2p* [4-1]

pCR185 GFP and *unc-54* 3’UTR [2-3]

pEGB05 *rab-3p* [4-1]

pET36 mouse *Eipr1* cDNA [1-2]

pGH1 *unc-17p* [4-1]

pMA78 *vps-52p* [4-1]

pMA79 *vps-53p* [4-1]

pMA80 *vps-53(+)* genomic [1-2]

pMA88 *vps-52(+)* genomic [1-2]

pSD12 *unc-129p* [4-1]

pT19D7.4[4-1] *rund-1p* [4-1]

pW09C5.1[4-1] *W09C5.1p* [4-1]

*eipr-1(+)* [1-2] *eipr-1(+)* genomic [1-2]

*eipr-1*[1-2]cDNA *eipr-1* cDNA [1-2]

Gateway expression constructs

pAP3 *W09C5.1p::GFP*

pAP5 *rab-3p::eipr-1 cDNA::GFP*

pET11 *unc-17p:: eipr-1 cDNA::GFP*

pET13 *unc-17Hp::eipr-1 cDNA::GFP*

pET14 *rab-3p::eipr-1 cDNA::GFP*

pET15 *acr-2p:: eipr-1 cDNA::GFP*

pET37 *rab-3p::mEipr1 cDNA::GFP*

pET110 *unc-129p::eipr-1 cDNA::GFP*

pMA77 *rund-1p::eipr-1(+)::tagRFP*

pMA82 *vps-52p::citrine*

pMA83 *vps-53p::citrine*

pMA84 *vps-53p::vps-53(+)::tagRFP*

pMA85 *rab-3p::vps-53(+)::tagRFP*

pMA89 *vps-52p::vps-52(+)::tagRFP*

Mammalian expression vectors and constructs

pEGFP-N1 Mammalian expression vector for EGFP tagging (Addgene, https://www.addgene.org/vector-database/2491/)

pmCherry-N1 Mammalian expression vector for mCherry tagging (gift from S. Hoppins)

pET42 rat *Eipr1* cDNA in pEGFP-N1

pET50 rat *Cccp1* cDNA in pEGFP-N1

pET55 rat *Rab2A* cDNA in pEGFP-N1

pET106 rat *Vps51* cDNA in pmCherry-N1
